# Robotic Surgery: A Narrative Review

**DOI:** 10.7759/cureus.29179

**Published:** 2022-09-15

**Authors:** Sakshi Bramhe, Swanand S Pathak

**Affiliations:** 1 Community Medicine, Jawaharlal Nehru Medical College, Datta Meghe Institute of Medical Sciences, Wardha, IND; 2 Pharmacology, Jawaharlal Nehru Medical College, Datta Meghe Institute of Medical Sciences, Wardha, IND

**Keywords:** urosurgery, robotic surgical training, telemanipulator systems, single-incision surgery, robotic urology, robotic surgery, robotic endoscopy, robot-assisted laparoscopy

## Abstract

In general surgery, the use of robotic and laparoscopic methods has increased. Robotic surgery that requires the least incision has advanced over the years in a short period of time, benefitting both the patient and the surgeon. According to this, robotic platforms and tools are now being used and improved more commonly in general surgery. In a quickly growing and dynamic environment of research and development, the goal of this review is to explore the present and emerging surgical robotic technologies. Future progress in robotics will focus primarily on more durable haptic systems that would provide tactile and kinesthetic input, miniaturisation and micro-robotics, better visual feedback with higher fidelity detail and magnification, and autonomous robots. It is recommended to develop a structured training course with benchmarks for success and evidence-based training strategies. This usually includes a step-by-step progression starting with observation, case aid in programming and manipulation of surgical instruments, learning the basics of robotics in a dry and wet lab setting, attaining non-technical skills on an individual and team level, and monitored modular console training, accompanied by autonomous practice. Prior to independent practice, basic robotics skills and procedural activities must be performed safely and effectively as part of robotic surgical training. It is advised to create a systematic training programme with performance indicators and research-based instructional techniques.

## Introduction and background

Technology has significantly changed surgical procedures, necessitating the development of new techniques to evaluate how successfully they were performed, particularly in the technical, legal and bioethical fields. The relationship between humans and machines has given rise to various ethical problems that demand a more serious evaluation. Although they have been illustrated in Isaac Asimov's science fiction works, in which they were part of his technophilic and arrogant vision of the future, in which robots would be a great help to humankind in the upcoming generations. This is because robots will become more common in daily life, performing tasks and helping people at home and at work, to become true "companions" of people in the not-too-distant future. Consider BINA48, a robot that is capable of understanding, interacting with humans, and expressing emotions. Robotic surgery pushes the boundaries of technical innovation in healthcare in the pursuit of better clinical results. We talk about the advancement of five generations of robotic surgical platforms including stereotactic, endoscopic, bioinspired, microbots on the millimetre scale, and the future creation of autonomous systems. By implementation of anatomical and immune-histological imaging, data assimilation, improved visualization, haptic feedback, and robot-surgeon interface, we examine the challenges, disadvantages, and potential of robotic surgery. In the surgical and anaesthetic journey, we take into account the existing data, cost-effectiveness, and learning curve, as well as what is necessary to continue to achieve improvements in surgical operative care. This technology's novel impacts have the potential to produce core clinical improvements [1].

## Review

"First Law: A robot may not injure a human being or, by omission, cause harm to a human being; Second Law: A robot must take human instructions unless they would conflict with the First Law; Third Law: The existence of a robot must be protected as long as it does not conflict with the First or Second Law." Robotic surgery implementation is commonly done in the surgical community to an incredible level. It has been fueled in part by the quick rise of technology and in part by how quickly and easily current laparoscopic procedures have been modified. The use of robots in medical procedures is quickly replacing human doctors. Similar to certain other technological advances in medicine and surgery, these developments have rarely been used as a result of randomised prospective study [2].

There are now three primary categories of robotic surgical systems in use: systems that are active, semi-active, and master-slave. While still being managed by the surgical team, active systems essentially operate autonomously and carry out pre-programmed tasks. Great examples of this are the platforms PROBOT and ROBODOC which are discussed later. With semi-active systems, the already programmed component of these robotic equipment can be complemented by a surgeon-driven component. The da Vinci® and ZEUS platforms (Intuitive Surgical, Sunnyvale, CA, USA) served as the prototypes for formal master-slave systems, which are free of any pre-programmed or autonomous features [2]. Although the early active robotic systems clearly illustrated the potential of mechanical devices to improve surgical interventions, the concept of telepresence, which resulted from a partnership between researchers from Stanford and NASA's Ames Research Centre, served as the catalyst for progressions that ultimately improved laparoscopic procedures. When the potential value of connecting doctors (far from the war) to patients via a robotic platform was realized, the US military found a possibility to reduce the morbidity and mortality of service members serving in areas of conflict. Many of the researchers who were initially linked to this military interest grouping moved on to market their ideas in the public and private sectors [2]. Man has been dreaming of the ability to reproduce himself using a mechanical robot construction for the past 3000 years. Robotics in medicine, meanwhile, has only been used for 30 years. Robotic surgery is a result of modern man's demand to achieve two things: telepresence and the execution of precise, repetitive tasks. The PUMA 200 was the first "robot surgeon" to operate on a human patient in 1985. Scientists created the "master-slave" robot concept in the 1990s, which involved a robot with remote manipulators that could be controlled by a surgeon at a surgical workstation [3]. However, increased cost and a lack of haptic input are the main obstacles preventing present robotic technology from becoming the standard for minimally invasive surgery everywhere in the globe. Therefore, lowering the costs, establishing and testing curriculum and virtual simulators, performing randomized clinical studies to determine the best applications of robotics, and introducing new platforms and technologies are all crucial elements of the future of robotic surgery [3].

Table 1 shows turnover (in billion USD) of all the service robots that are used for professional use, this includes medical robots, logistics robots, and field robots. All the possible turnovers that occurred during the years 2018, 2019, 2020, 2021, 2022, and 2023 are shown in the given table.

**Table 1:** Service robots for professional use: Turnover 2018, 2019, potential development 2020-2023 (in billion USD)

	Medical robots	Logistics robots	Field robots
2018	4.1	0.9	1.3
2019	5.3	1.9	1.3
2020	6.6	2.6	1.4
2021	8.6	3.7	1.6
2022	11.3	5.3	1.8
2023	12.6	7.5	2.0

Surgical intervention and surgical practice have undoubtedly changed as a result of robotic surgery. Currently, a variety of platforms are used to carry out a wide range of procedures, with varied degrees of success and applications. Surgeons and the medical community reported better outcomes with this procedure compared to conventional laparoscopy due to a number of technological advancements, including graphic designing due to the three-dimensional interface, vibration filtration, continued to improve wrist motion freedom, motion scalability, and enhanced ergonomics due to a more pleasant user interface. Today's procedures and specialties use robotic surgical systems, including cardiology, urology, endocrinology, metabolic, and bariatric surgery, head and neck surgery, and all intra-abdominal surgical subspecialties [4].

Urologists' use of robotic surgery has been followed by a growing understanding of the importance of formal training. Early research on robotic surgery showed the future function of this technology to be uncertain. A study of 372 residents and 56 program directors conducted in 2006 found a little experience and a great deal of ambiguity regarding the current use of robotics. A little over 15% thought robotics was a "trend that would fail," 35% said it was "here to stay," and 50% were "unsure." The majority of residents were either unsure (33%) or sceptical (30%) about doing robotic surgery in real-world situations. However, 74% of residents and program directors believed that the usage of robotic surgery would rise over the coming years. Similar percentages of graduates who expected to do robotic surgery in the future (between 29 and 39%) and who believed that the usage of robotic surgery would expand in the future were found in a 2007 and 2008 study of 56 senior urology residents in Canada (71%-75%) [5]. The current definition of robotic surgery calls for the use of tiny wristed tools connected to a robotic arm to carry out surgical procedures. The surgeon controls the device to achieve high-definition magnification while utilizing the precision and miniaturisation abilities of the robotic arm. Robots were first used in medical applications 30 years ago, and today they are one of the surgical field's fastest-growing segments. For head and neck surgeons, transoral robotic surgery (TORS) has emerged as a useful and secure technology [6].

**Figure 1:**
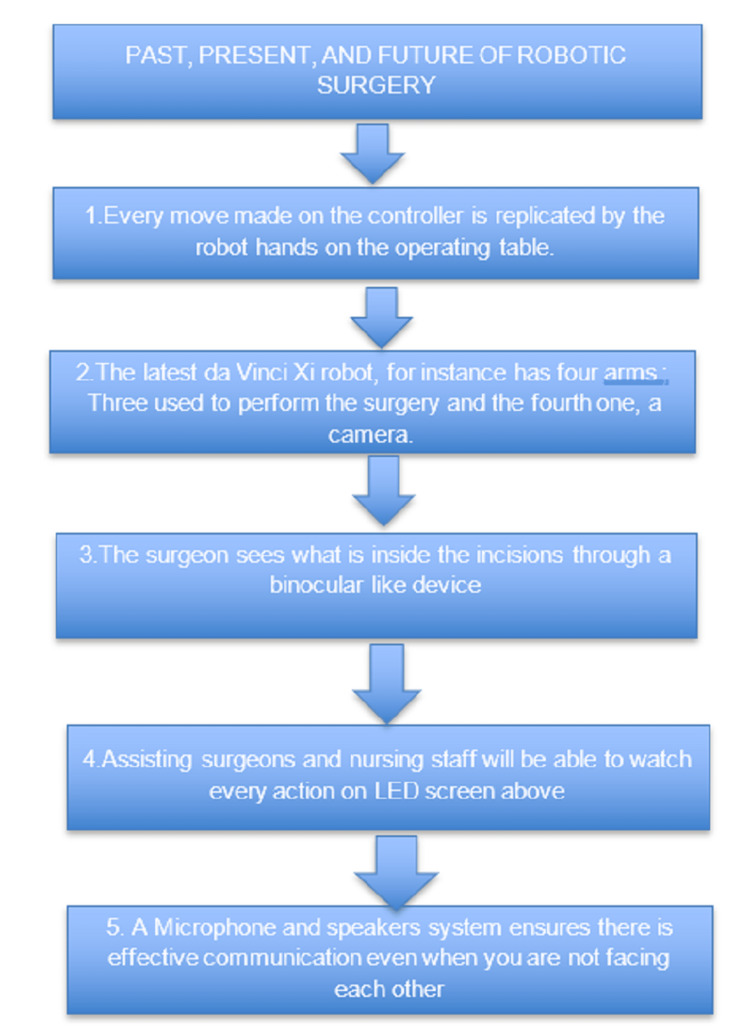
Robotic surgery: how it works

Figure 1 shows the steps of how robotic surgery is evolved. The past, present and future of robotic surgery are shown in five steps. This includes the use of a controller, the latest da Vinci Xi robot (Intuitive Surgical, Sunnyvale, CA, USA) that has four arms, the use of each arm is specified, usage of the binocular device, and the ability to watch those surgeries on screen, also the use of a microphone is shown to have proper communication.

The way that traditional mechanical robots work is by relaying the motions of the surgeon's hands to the surgical target through the vibration movements of surgical tools. Similar to this, future generations of surgical robots adapt human-initiated activities to a tailored surgical plan by utilizing 3D digital segmentation created before surgery. Research and development of intelligent robots have risen across all facets of human life as a result of advances in cloud computing, big data analytics, and artificial intelligence. Several surgical firms are collaborating with tech behemoths to create intelligent surgical robots, spurred on by the successful use of deep learning. To develop, specify, and deploy deep learning models for creating autonomous robots, we will here highlight important phases in the processing and analysis of massive data [7]. In comparison to laparoscopic surgery, the use of robotic-assisted surgery increased 10 to 40 times in typical general surgical procedures in 2017. A consistent training program is required due to the quick rise in the usage of robotic-assisted surgery. Numerous training programs are being created and tested right now. Furthermore, improvements in the use of virtual simulation instruments have made it easier to include them in training for robotic surgery. This review intends to highlight, analyze, and provide updates on the relevant validation processes for the existing curriculum and robotic-assisted surgery training simulators [8].

Robotic head and neck surgery makes use of the specific anatomy and built-in entry points for surgery to apply minimally invasive concepts. Surgical robotics has revolutionized head and neck surgery, expanding on a heritage of minimally invasive endoscopic otolaryngology procedures. Anatomical restrictions impair visibility and limit surgical operations, nevertheless, surgeons must overcome major obstacles [9]. Although there are numerous potential benefits to using a robotic minimally invasive technique, doing so requires a high level of technical knowledge and experience, particularly in some of the intricate and technically difficult situations that are frequently seen in first referral units (FRU). In addition to the surgical concepts of FRU, surgeons should make sure they have received adequate training in patient assessment, treatment, and follow-up. Additionally, it is crucial to avoid important surgical measures [10]. Within this quickly developing discipline, paediatric robotic surgery poses special obstacles. For what are essentially low-volume complex instances, delivering this cutting-edge technology comes with a hefty price tag. Most paediatric surgical centres all around the world have only slowly begun to use this technology due to the cost of purchasing equipment primarily intended for adults as well as financial constraints. The majority of paediatric surgery centres throughout the world have had a slow rate of acceptance as a result. Little kids' limited workspace is now a problem due to the ergonomics needed for the da Vinci® master-slave platform [11].

Robots intended for eye surgery should adhere to a few fundamental standards. The three designs now being developed are the steady hand, co-manipulation tools, and telemanipulators using either a fixed or virtually distant centre of the action. Surgical technique is being used to successfully operate on human eyes. Despite its nonophthalmic design, the da Vinci Surgical System, another telemanipulation robot, proved successful in ex-vivo corneal surgery and has been used to successfully correct pterygia in people [12]. Transoral robotic surgery has developed from an experimental design to becoming widely accepted in the treatment of head and neck tumours and other disease states over the course of nearly a decade, from 2005 to 2015. It has been well established that transoral robotic surgery is used to treat laryngeal and pharyngeal cancer. The secret to widespread use and acceptance was education and training [13].

Robotic surgery has entered the arsenal of pelvic organ prolapse (POP) treatment, enabling pelvic surgeons to modify the "gold standard" abdominal sacral technique to a minimally invasive method with reduced postoperative convalescence. In reality, robotic surgery can be used to correct pelvic organ prolapse, and sometimes surgeons find that the robot's aid makes suturing and dissection during the procedures less difficult. Even though robotic surgery may have several advantages over traditional laparoscopy, these benefits should still be compared to the expense of the technology [14]. The advantages of minimally invasive surgery are maintained while total mesorectal excision treatments for rectal cancer are made simpler using advanced robotic technology. In comparison to conventional laparoscopic surgery, robotic surgery for rectal cancer has reduced conversion rates and hastens the return of urogenital function. The two biggest drawbacks of robotic surgery are its extended operating times and hefty costs [15].

The use of laparoscopic surgery as a less invasive method of treating colon cancer has gained recognition. However, due to the limited accessibility, laparoscopic surgery used for rectal cancer, especially lower rectum cancer, is still difficult. Robotic surgery provides benefits such as 3D imaging, dexterity and ambidextrous ability, lack of vibration, scaling of motion, and a quick learning curve. It also overcomes the anatomical restrictions of laparoscopy [16]. Robotic colorectal surgery is becoming more and more common, and both patients and surgeons have reportedly benefited from it. Although there is strong evidence in favour of its usage for pelvic surgery, there is less proof that it is advantageous for abdominal surgery. Robotic surgery has several technical restrictions that have been overcome by newer generations of robotic platforms, which means that its use will likely rise in the near future [17]. But the surgical sector has been utilizing these newly developed technologies, particularly the employment of contemporary robotic systems made up of a vision device and a motor device (for which surgical tools are responsible). In some circumstances, there is an additional vocal command system that makes the work of the surgeon easier to manage the device [18]. There have been a few transoral robotic surgical procedures performed on the anterior skull base, but there are still some significant restrictions to its application there. The size of the robotic endoscope and the equipment that is now available is the main constraint. Robotic anterior skull base surgery holds great promise for the future thanks to technological developments including single-port robots, flexible instrument systems, and the miniaturisation and expansion of minimally invasive platforms [19, 20].

In colorectal surgery, laparoscopy lowers the incidence of postoperative problems, shortens hospital stays, and enhances patient care. Despite these well-established advantages, the majority of surgeries are still carried out by open surgery in the USA due to the technical difficulties of rectal resection for cancer [21]. Furthermore, for some patient-reported outcome criteria, such as scar cosmesis and discomfort, robotic thyroidectomy has been proven to be an upper hand over open surgery. Its disadvantages include high cost, extended operating time, and potential for problems not present in open thyroidectomy [22].

Robotic simulation is still developing and augmented reality is already a part of robotic surgical education because of advancements in the simulation of training design. Robotic-assisted laparo-endoscopic single-site surgery is still developing, and advancements in technology now make it possible to handle pathologic surgical anatomy that was previously difficult, such as urologic oncology and reconstruction [23]. Especially in North America and Europe, robot-assisted laparoscopic surgery (RALS) has grown in popularity in the disciplines of adult and paediatric urology. Tremor filtering, motion scaling, and a stable enlarged 3D image are benefits that enable accurate intra-corporeal exposure and suturing. The robotic platform is highly suitable for the field of child urology because the bulk of operations is performed as reconstructive rather than excision procedures [24]. When parathyroid carcinoma or adenoma has been pre-operatively localized, robotic parathyroidectomy is a novel surgical method in the treatment of primary hyperparathyroidism. It represents the "fourth generation" in the evolution of parathyroid surgery after a process of surgical evolution from cervicotomy and 4-gland exploration to a variety of minimally invasive, open and endoscopic, targeted approaches [25]. With ideas for improving and sustaining pertinent frameworks or standards, we address the specific challenges for robotic surgery presented by proposals made for AI more generally (e.g., explainable AI) and machine learning more specifically (e.g., black box) [26]. However, many of Intuitive Surgical earliest patents would expire in the following many years due to the complex nature of US patenting law. With such a change in the environment, many of Intuitive Surgical rivals (with backgrounds in industrial and medical robotics) have started the robotic program, some of which are currently useful in clinical settings [27]. Modern medicine largely depends on the capacity to do minimally invasive surgery, thus laparoscopic tools and techniques are constantly evolving. Over time, the shift from open surgery to minimally invasive surgery led to the introduction of robotic tools to either enhance or entirely replace patient-side surgery with a separate console [28]. While there are numerous reports on robotic bariatric surgery that are currently being released, the bulk of these studies has weak supporting information. As a result, even though robotics technology is undoubtedly better than traditional laparoscopy, it is still unclear exactly what role robots will play in bariatric surgery [29]. Robotic surgical and medical platforms have become more widespread, and technology is increasingly being developed to meet this demand. Even more, technologies are being used to increase the capabilities of existing systems. Research is still needed to assess the benefits and disadvantages of each robotic surgical tool and base used in the operation room [30]. Patients with head and neck cancer may benefit from robotic surgery, and in some cases, it may even be more efficient than regular procedures in terms of cosmetic, functional, and oncological results [31].

The robotic platform's special abilities have made minimally invasive surgery viable and safe with outcomes that are on par with, if not better than, those of open surgery (e.g., shorter length of stay, fewer infections in surgical site) [32]. It is recommended to develop a structured training course with benchmarks for success and evidence-based training strategies. This usually includes a step-by-step progression starting with observation, case aid, learning the foundations of robotics in a dry and wet lab setting, achieving non-technical skills on an individual and team level, and modular training which is done under supervision and is independent [33]. The invention of robotic surgery is one of the most important inventions, which has made even the most complex treatments possible through the articulation of the instruments and the precision of movement [34]. Programs like ours can benefit from the organization of a centre of excellence in robotic surgery, which will decrease complications and effects produced. This centre will be made up of certified robotic surgical experts from each speciality and hospital administrators for the accreditation of standards for both the initial and re-certification of their doctors [35]. Future progress in robotics will focus primarily on more lasting haptic systems that would provide tactile and kinesthetic input, downsizing and micro-robotics, improved visual feedback with higher fidelity detail and magnification, and autonomous robots [36].

Long-term results affect a robotic surgery program's viability and durability. To build a successful program, meticulous planning with the robotics committee, outlining the kinds of procedures performed and sufficient multidisciplinary training to prevent surgical cancellations, is essential [37]. Even though only one robotic surgery for the colonic disease has seen significant advancements in the design and modification of solitary ports, robotic arm combinations, and robotic surgical platforms, there still seems to be a lot of work to be done on these platforms [38]. Many aspects of conducting complex surgeries have been transformed by the robotic surgical technology that has emerged during the past 15 years. It combines technical and clinical innovations to enhance the calibre of surgery and patient outcomes. The training and certification of robotic surgeons, however, are still not standardized [39]. In a highly competitive environment, surgery can only continue to play a role if outcomes are consistently improved, along with a continued decrease in trauma for patients and with reasonable costs. However, a true breakthrough has not yet been made. Minimally invasive surgery, and particularly robotic surgery, is expected to provide a significant push toward achieving this goal [40].

## Conclusions

The influence of robots in modern life has grown since they first emerged in the literature. In reality, you may observe them operating in locations where people can't due to biological limitations and assisting both men and women in numerous science sectors, such as the study of health. In this context, the emphasis is on how these devices have advanced in their use during surgical procedures, producing beneficial results across a range of therapies. In this case, the bioethical discussion of robotic surgery - which is still in its developing stage in educational circles and medical research - becomes very helpful in supporting decision-making when robots are engaged in delivering care to humans. Based on our first experiences, we would suggest a training roadmap that is also outlined with assessments of ability at each level before progression. The foundation of such a training course would be the acquisition of non-technical abilities in the practising lab before moving on to modular training and concluding with sign-off and autonomous practice. A demonstration of competence and safety in carrying out fundamental robotic abilities and procedural tasks would also be necessary.

## References

[1] Ashrafian H, Clancy O, Grover V, Darzi A (2017). The evolution of robotic surgery: surgical and anaesthetic aspects. Br J Anaesth.

[2] Lane T (2018). A short history of robotic surgery. Ann R Coll Surg Engl.

[3] Leal Ghezzi T, Campos Corleta O (2016). 30 years of robotic surgery. World J Surg.

[4] Jara RD, Guerrón AD, Portenier D (2020). Complications of robotic surgery. Surg Clin North Am.

[5] Wang RS, Ambani SN (2021). Robotic surgery training: current trends and future directions. Urol Clin North Am.

[6] Maza G, Sharma A (2020). Past, present, and future of robotic surgery. Otolaryngol Clin North Am.

[7] Bhandari M, Zeffiro T, Reddiboina M (2020). Artificial intelligence and robotic surgery: current perspective and future directions. Curr Opin Urol.

[8] Chen R, Rodrigues Armijo P, Krause C, Siu KC, Oleynikov D (2020). A comprehensive review of robotic surgery curriculum and training for residents, fellows, and postgraduate surgical education. Surg Endosc.

[9] Finegersh A, Holsinger FC, Gross ND, Orosco RK (2019). Robotic head and neck surgery. Surg Oncol Clin N Am.

[10] Osman NI, Mangir N, Mironska E, Chapple CR (2019). Robotic surgery as applied to functional and reconstructive urology. Eur Urol Focus.

[11] Cave J, Clarke S (2018). Paediatric robotic surgery. Ann R Coll Surg Engl.

[12] de Smet MD, Naus GJ, Faridpooya K, Mura M (2018). Robotic-assisted surgery in ophthalmology. Curr Opin Ophthalmol.

[13] Thaler ER (2020). History and acceptance of transoral robotic surgery. Otolaryngol Clin North Am.

[14] Giannini A, Russo E, Malacarne E, Cecchi E, Mannella P, Simoncini T (2019). Role of robotic surgery on pelvic floor reconstruction. Minerva Ginecol.

[15] Baek SJ, Piozzi GN, Kim SH (2021). Optimizing outcomes of colorectal cancer surgery with robotic platforms. Surg Oncol.

[16] Nozawa H, Watanabe T (2017). Robotic surgery for rectal cancer. Asian J Endosc Surg.

[17] Pai A, Marecik S, Park J, Prasad L (2017). Robotic colorectal surgery for neoplasia. Surg Clin North Am.

[18] Siqueira-Batista R, Souza CR, Maia PM, Siqueira SL (2016). Robotic surgery: bioethical aspects. Arq Bras Cir Dig.

[19] Campbell RG, Harvey RJ (2021). How close are we to anterior robotic skull base surgery?. Curr Opin Otolaryngol Head Neck Surg.

[20] Zheng HX, Cheng JW (2020). Standardized training in robotic surgery in urology and andrology [Article in Chinese]. Zhonghua Nan Ke Xue.

[21] Achilli P, Grass F, Larson DW (2021). Robotic surgery for rectal cancer as a platform to build on: review of current evidence. Surg Today.

[22] Aidan P, Arora A, Lorincz B, Tolley N, Garas G (2018). Robotic thyroid surgery: current perspectives and future considerations. ORL J Otorhinolaryngol Relat Spec.

[23] Gettman M, Rivera M (2016). Innovations in robotic surgery. Curr Opin Urol.

[24] Mizuno K, Kojima Y, Nishio H, Hoshi S, Sato Y, Hayashi Y (2018). Robotic surgery in pediatric urology: current status. Asian J Endosc Surg.

[25] Arora A, Garas G, Tolley N (2018). Robotic parathyroid surgery: current perspectives and future considerations. ORL J Otorhinolaryngol Relat Spec.

[26] O'Sullivan S, Nevejans N, Allen C (2019). Legal, regulatory, and ethical frameworks for development of standards in artificial intelligence (AI) and autonomous robotic surgery. Int J Med Robot.

[27] Brodie A, Vasdev N (2018). The future of robotic surgery. Ann R Coll Surg Engl.

[28] Williamson T, Song SE (2022). Robotic surgery techniques to improve traditional laparoscopy. JSLS.

[29] Jung MK, Hagen ME, Buchs NC, Buehler LH, Morel P (2017). Robotic bariatric surgery: a general review of the current status. Int J Med Robot.

[30] Peters BS, Armijo PR, Krause C, Choudhury SA, Oleynikov D (2018). Review of emerging surgical robotic technology. Surg Endosc.

[31] Burton J, Wong R, Padhya T (2015). Robotic-assisted surgery in the head and neck. Cancer Control.

[32] Joyce D, Morris-Stiff G, Falk GA, El-Hayek K, Chalikonda S, Walsh RM (2014). Robotic surgery of the pancreas. World J Gastroenterol.

[33] Sridhar AN, Briggs TP, Kelly JD, Nathan S (2017). Training in robotic surgery-an overview. Curr Urol Rep.

[34] Rumolo V, Rosati A, Tropea A, Biondi A, Scambia G (2019). Senhance robotic platform for gynecologic surgery: a review of literature. Updates Surg.

[35] Vásquez-Lastra C, Decanini-Terán C, Maffuz-Aziz A (2021). Robotic surgery at ABC Medical Center: first 500 procedures experience. Gac Med Mex.

[36] Alip SL, Kim J, Rha KH, Han WK (2022). Future platforms of robotic surgery. Urol Clin North Am.

[37] Giedelman C, Covas Moschovas M, Bhat S (2021). Establishing a successful robotic surgery program and improving operating room efficiency: literature review and our experience report. J Robot Surg.

[38] Bae SU, Jeong WK, Baek SK (2017). Current status of robotic single-port colonic surgery. Int J Med Robot.

[39] Andolfi C, Umanskiy K (2017). Mastering robotic surgery: where does the learning curve lead us?. J Laparoendosc Adv Surg Tech A.

[40] Feußner H, Wilhelm D (2016). Minimally invasive surgery and robotic surgery: surgery 4.0? [Article in German]. Chirurg.

